# Cis-regulatory signatures of orthologous stress-associated bZIP transcription factors from rice, sorghum and Arabidopsis based on phylogenetic footprints

**DOI:** 10.1186/1471-2164-13-497

**Published:** 2012-09-20

**Authors:** Fuyu Xu, Myoung-Ryoul Park, Ai Kitazumi, Venura Herath, Bijayalaxmi Mohanty, Song Joong Yun, Benildo G de los Reyes

**Affiliations:** 1 School of Biology and Ecology, University of Maine, 5735 Hitchner Hall, Orono, ME, 04469, USA; 2 Department of Chemical and Biomolecular Engineering, National University of Singapore, Singapore, Singapore, 117576; 3 Department of Crop Science and Institute of Agricultural Science and Technology, Chonbuk National University, Chonju, 561-756, Korea; 4 Present Address: Department of Agricultural Biology, Faculty of Agriculture, University of Peradeniya, Peradeniya, Sri Lanka, 20400

**Keywords:** Orthologs, Cis-elements, Stress, bZIP transcription factor, Phylogenetic footprinting

## Abstract

**Background:**

The potential contribution of upstream sequence variation to the unique features of orthologous genes is just beginning to be unraveled. A core subset of stress-associated bZIP transcription factors from rice (*Oryza sativa*) formed ten clusters of orthologous groups (COG) with genes from the monocot sorghum (*Sorghum bicolor*) and dicot Arabidopsis (*Arabidopsis thaliana*). The total cis-regulatory information content of each stress-associated COG was examined by phylogenetic footprinting to reveal ortholog-specific, lineage-specific and species-specific conservation patterns.

**Results:**

The most apparent pattern observed was the occurrence of spatially conserved ‘core modules’ among the COGs but not among paralogs. These core modules are comprised of various combinations of two to four putative transcription factor binding site (TFBS) classes associated with either developmental or stress-related functions. Outside the core modules are specific stress (ABA, oxidative, abiotic, biotic) or organ-associated signals, which may be functioning as ‘regulatory fine-tuners’ and further define lineage-specific and species-specific cis-regulatory signatures. Orthologous monocot and dicot promoters have distinct TFBS classes involved in disease and oxidative-regulated expression, while the orthologous rice and sorghum promoters have distinct combinations of root-specific signals, a pattern that is not particularly conserved in Arabidopsis.

**Conclusions:**

Patterns of cis-regulatory conservation imply that each ortholog has distinct signatures, further suggesting that they are potentially unique in a regulatory context despite the presumed conservation of broad biological function during speciation. Based on the observed patterns of conservation, we postulate that core modules are likely primary determinants of basal developmental programming, which may be integrated with and further elaborated by additional intrinsic or extrinsic signals in conjunction with lineage-specific or species-specific regulatory fine-tuners. This synergy may be critical for finer-scale spatio-temporal regulation, hence unique expression profiles of homologous transcription factors from different species with distinct zones of ecological adaptation such as rice, sorghum and Arabidopsis. The patterns revealed from these comparisons set the stage for further empirical validation by functional genomics.

## Background

Regulatory transcription factors are among the major forces that drive the evolution of multicellular complexity. As such, they represent a group of highly conserved network hubs that directly link gene expression programs to various internal and external signals for development, growth and adaptation [1]. Changes in the regulation of these network hubs lead to a ‘network rewiring effect’, which is manifested by dynamic changes in transcriptome and proteome signatures [2-4]. Indeed, much of the physiological and developmental variations across the evolutionary continuum of the plant kingdom are to a large extent consequences of how regulatory transcription factors have been reprogrammed over time to create diverse network configurations [5-7].

The separation of the monocot and eudicot lineages of flowering plants spans more than 140 million years of evolutionary history [8]. Comparative analysis of reference genome sequences across a number of representatives from the monocot and eudicot lineages revealed the dynamic changes in genome size, organization and complexity during speciation [9-12]. Gene duplication is a hallmark of such dynamic changes in genome complexity [13]. For regulatory transcription factors, the function of duplicated copies may exhibit various degrees of conservation and divergence at different taxonomic levels. The degree of functional divergence may be apparent not only in terms of structure but most importantly in terms of differences in spatio-temporal programs, some of which may have important implications to a fine-tuned physiological process integral to the adaptive properties of a given species to its ecological niche [14].

Recent studies comparing the evolution of genetic network complexities in flowering plants revealed important roles of regulatory transcription factor evolution to physiological variation among species [6,15,16]. Large gene families tend to exhibit greater functional diversity as various members are often co-opted for more specialized biological roles. The transcriptional regulators (*CO* and *FT)* of the flowering response pathway in the eudicot plant *Arabidopsis thaliana* have functional homologs (*Hd1* and *Hd3a,* respectively) in the monocot plant rice (*Oryza sativa*). The diurnal expression patterns are remarkably conserved between the Arabidopsis and rice homologs through the specific functions of their respective phytochrome chromophore proteins (*HY1* in Arabidopis and *SE5* in rice). However, similarities in expression are punctuated by changes in other features that serve to fine-tune physiological functions in the divergent species, while maintaining the broad biological function of the gene. *CO* evolved to function as an activator that promotes the expression of *FT* to trigger floral development of Arabidopsis under long days, while *Hd1* functions as a repressor that prevents *Hd3a* expression, keeping the rice plant in vegetative phase under long days [17,18]. The co-option scenario is illustrated by numerous examples of transcription factor families (e.g., MADS, bHLH, NAC, ERF) that have undergone significant expansion during speciation leading to lineage-specific functions for both developmental and stress-related responses [1,7,15,19].

The relationships among ‘equivalent’ transcription factors in divergent plant species are traditionally expressed in terms of homologous clusters whose members are likely to have evolved from a common ancestral gene. Under each cluster are the orthologous genes whose functions are conserved after speciation, and the paralogous genes whose functions have become more specialized being the outcomes of gene duplication [20]. Conventionally, orthologs are established by one-to-one or many-to-many relationships through phylogenetic reconstructions based on coding sequences and conservation of protein domains [21].

Identification of regulatory transcription factors on the basis of orthologous relationship facilitated a meaningful translation of knowledge regarding biological function from model to non-model species. While such knowledge contributed immensely to the strength of the comparative functional genomics paradigm, understanding the broad biological function of transcription factors in an evolutionary context is not complete without a systematic investigation of the contribution of upstream sequence variation to the potentially unique regulatory features of each member of an orthologous group. In stress physiology, such information could potentially define another layer of complexity and might open a different view of why different species have distinct ecological niches despite their largely homologous gene sets involved in various stress responses.

The bZIP-type transcription factors belong to a relatively large family of regulators with major roles in abiotic and biotic stress response mechanisms and in seed development and maturation, primarily through the ABA signal transduction pathway [22-25]. In our previous study, we have identified a subset of bZIP transcription factors involved in the regulation of low temperature response transcriptome of temperate rice, *Oryza sativa* ssp. japonica cv. Nipponbare [26]. We proposed that this group functions broadly in various stress regimes by virtue of their linkages with oxidative-mediated genetic network that plays a key role in almost every type of abiotic and biotic stresses. Furthermore, we were particularly interested in understanding the extent by which function and regulation of these general stress response transcription factors are conserved across species representing distinct stress physiological categories.

In the follow-up study described here, the aforementioned subset of stress-associated bZIP transcription factors was examined further in an evolutionary context by assessing the similarities and differences in upstream regulatory information content of the rice (*Oryza sativa)* genes and their orthologs in sorghum (*Sorghum bicolor)* and Arabidopsis (*Arabidopsis thaliana)* by phylogenetic footprinting [27,28]. *Bona fide* upstream sequences based on alignment with full-length cDNAs are critical for establishing biologically meaningful trends from these analyses. The annotated reference sequences of rice, sorghum and Arabidopsis represent a robust comparative genomics system that is particularly suitable for such application. Furthermore, rice, sorghum and Arabidopsis represent a meaningful spectrum of diversity in the plant kingdom, encompassing the monocot and dicot divide with relevant stress physiological attributes. We discuss our interpretations of the possible implications of the patterns that we observed in these analyses within the context of the finer-scale differences that may likely confer unique regulatory attributes to each member of an orthologous group. With the window of resolution afforded by the rice-sorghum-Arabidopsis comparative genomics, we have set the stage for more strategic experimental validation of cis-regulatory signatures of orthologous stress-associated bZIP transcription factors by functional genomics.

## Results and discussion

### Stress-regulated bZIP transcription factors of japonica rice

**Figure 1:**
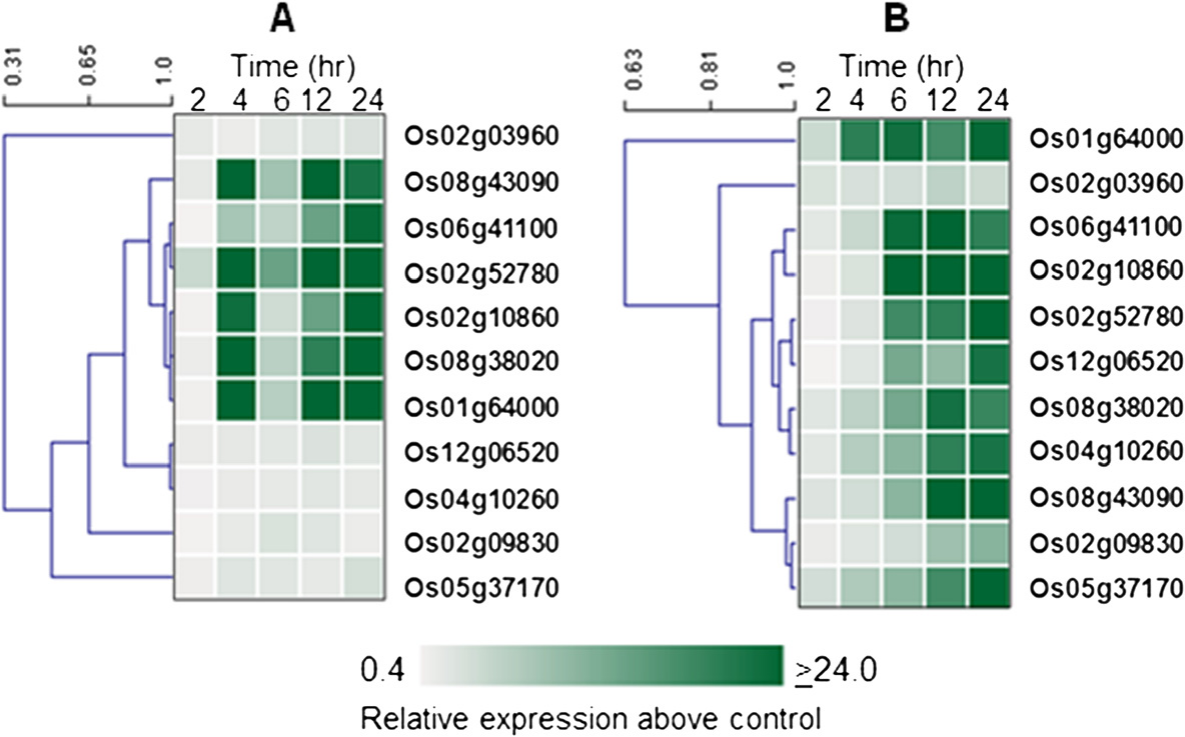
**Expression profiles of the core subset of low temperature and oxidative regulated bZIP transcription factors identified from a genome-wide survey in rice (*Oryza sativa*); (A) high salinity, (B) rapid dehydration.**

The total chilling stress transcriptome of japonica rice cv. Nipponbare includes 11 upregulated bZIP transcription factors described in earlier reports [23,26]. Ten of these genes were also upregulated by oxidative stress mimic based on induction patterns during exposure to 4 mM H_2_O_2_. Additionally, we found that six of the genes in this set (Os01g64000, Os02g10860, Os02g52780, Os06g41100, Os08g38020, Os08g43090) were also significantly upregulated by salinity (300 mM NaCl) and nine (Os01g6400, Os02g10860, Os02g52780, Os04g10260, Os05g37170, Os06g41100, Os08g38020, Os08g43090, Os12g06520) were also highly induced at different temporal patterns during rapid dehydration (Figure 1). Many of these genes are also involved in disease response mechanisms based on literature information and genome annotation [24,29,30]. These results indicate that the 11 transcription factors included in this study represent a core subset of the larger bZIP family with active roles in various stress response mechanisms.

### Orthologous groups of stress-associated bZIP genes in rice, sorghum and Arabidopsis

**Figure 2:**
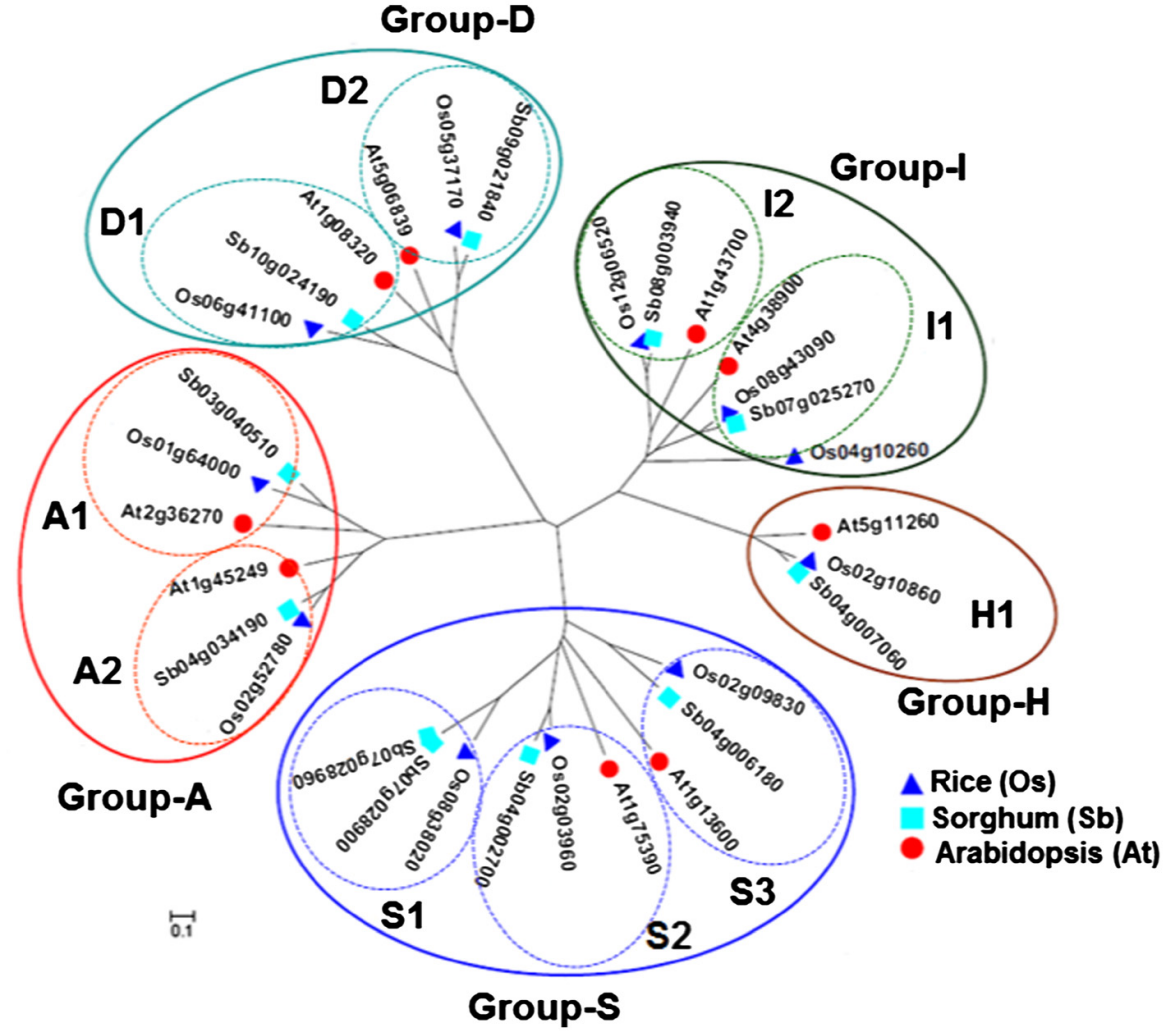
**Phylogenetic reconstruction of the orthologous stress-associated bZIP transcription factors of *Oryza sativa *(Os), *Sorghum bicolor *(Sb) and *Arabidopsis thaliana *(At).** The specific branches of the radial tree corresponding to the established functional sub-classes of bZIP transcription factors are indicated as Group-A, Group-D, Group-I, Group-H and Group-S [22]. Each group is comprised of clusters of orthologous groups (tri-species COG). Genes from the same species that belong to two neighboring COGs are considered paralogous to each other. A phylogenetic tree that includes other paralogs is shown in Additional file 1.

Using the 11 stress-related bZIP transcription factors of rice as queries, we performed parallel BlastN and BlastX searches of the annotated sorghum and Arabidopsis genomes in order to identify the best hit in each species and to establish orthologous and paralogous relationships [21,31-33]. Phylogenetic reconstructions based on amino acid sequence alignment and conserved bZIP domains further established the clear orthologs in the monocot sorghum and dicot Arabidopsis for ten of the eleven genes, forming ten clusters of orthologous groups (COGs) in either one-to-one or one-to-few relationships (Figure 2). Orthology was also consistent with available information from public databases such as the MSU Rice Genome Annotation Project and Gramene Comparative Grass Genomics Resources [34,35]. Involvement in stress response mechanisms of all orthologs from sorghum and Arabidopsis was also supported by published functional validation studies and/or by upregulated expression in response to one or more stress factors according to public genome annotation and expression databases [30,36-38].

Based on all available information, association with some kind of stress (abiotic and/or biotic) response mechanism was presumed to be the common function that defines the orthologous relationships among this panel of homologous bZIP transcription factors. Representative paralogs without an apparent association with stress response based on the rice expression data were also identified in all BlastN and BlastX searches and phylogenetic reconstructions set them apart from the clear orthologous gene sets (Additional file 1).

The COGs of the stress-associated bZIP transcription factors represent five functional sub-classes generally associated with ABA and stress response (Group-A), oxidative and pathogen defenses (Group-D), photomorphogenesis (Group-H), response to oxidative stress and gibberellic acid (Group-I) and stress response and sucrose signaling (Group-S) based on established functional classification schemes (Figure 2) [22,24]. Group-S is the largest group with two complete COGs with one member from each species (S2 = Os02g03960, Sb04g002700, At1g75390; S3 = Os02g09830, Sb04g006180, At1g13600) and one partial COG without a member from Arabidopsis but duplicated copies in sorghum (S1 = Os08g38020, Sb07g028900, Sb07g028960). Group-H is the smallest group with only one complete COG with one member from each species (H1 = Os02g10860, Sb04g007060, At5g11260). Both Group-A (A1 = Os016400, Sb03g040510, At2g36270; A2 = Os02g52780, Sb04g034190, At1g45249) and Group-D (D1 = Os06g41100, Sb10g024190, At1g08320; D2 = Os05g37170, Sb09g021840, At5g06839) have two complete COGs with one member from each species. Group-I includes two complete COGs with one member from each species (I1 = Os08g43090, Sb07g025270, At4g38900; I2 = Os12g06520, Sb08g003940, At1g43700) and one rice gene (Os04g10260) with no clear orthologs in sorghum and Arabidopsis.

### Total regulatory information content of stress-related bZIP COGs

The occurrence of 6 to 8 nucleotide sequence motifs representing putative transcription factor binding sites (TFBS) or cis-elements was first established in the upstream (−1,000 to +200) sequences of all orthologous and paralogous genes included in the study based on the plant-specific cis-element annotation in the Genomatix, TRANSFAC and PLACE promoter databases (Additional file 2). The TFBS classes that occurred among orthologs but not in paralogs were identified in order to establish the core subset of ortholog-specific cis-elements that could then be used to search for lineage-specific and species-specific conservation patterns. The core subset of ortholog-specific TFBS was also used to assess the differences in total cis-regulatory formation content between the different COGs and between the members of each COG. Total cis-regulatory information content represents all the motif classes in a given promoter with an e-value of 10^-3^ or less.

Table 1 summarizes the general trends in cis-regulatory information content for the representative COGs, showing all apparent patterns. Species-specific information content tends to be much higher than the COG-specific information content, providing a rough measure of the extent of sequence diversity between orthologous rice, sorghum and Arabidopsis promoters. Chi-square test for the equality of the number of cis-element classes detected in each promoter (1:1:1 or 1:1) across all three species and between any two given species in all possible comparisons (monocot vs. monocot, dicot vs. monocot) indicate that monocot and dicot orthologs had similar information content for A1 and A2 and all three orthologs had significantly different information content for S2. Differences in information content between monocot and dicot orthologs are most apparent for D1, D2, I1 and I2. Overall, monocot orthologs tend to be more similar to each other than to their dicot counterpart as revealed by similar information content for the rest of the COGs except H1.

**Table 1:** Total cis-regulatory information content of orthologous stress-associated bZIP transcription factors of rice, sorghum and Arabidopsis

**Tri-species COG**									
**Comparison**	**A1**	**A2**	**D1**	**D2**	**H1**	**I1**	**I2**	**S1**	**S2**
TFBS in all species	23	12	18	20	22	23	17	18	10
TFBS in Os only	65	45	74	39	85	70	52	69	38
TFBS in Sb only	70	42	70	48	62	70	44	62	17
TFBS in At only	83	48	99	91	62	92	60	81	63
*X*^2^ (Os:Sb:At) = 1:1:1	2.357	0.400	**6.099**	**26.015**	**5.061**	**4.173**	2.462	**3.911**	**26.927**
			**	****	**	**		**	****
*X*^2^ (Os:Sb) = 1:1	0.185	0.182	0.111	0.931	**3.599**	0.000	0.667	0.374	**8.018**
					*				***
*X*^2^ (Os:At) = 1:1	2.189	0.043	**3.613**	**20.800**	**3.599**	**2.988**	0.571	0.960	**6.188**
			*	****	*	*			**
*X*^2^ (Sb:At) = 1:1	1.105	0.400	**4.976**	**13.302**	0.000	**2.988**	**2.762**	2.524	**26.450**
			**	****		*	*		****

**Probability:** 2.706 (α = 0.1)*; 3.841 (α = 0.05)**; 6.635 (α = 0.01)***; 10.827 (α = 0.001)****.

Rice (Os); Sorghum (Sb); Arabidopsis (At).

### General patterns of cis-regulatory conservation among COGs

To establish the common denominator among the members of the tri-species COGs with respect to cis-regulatory information content, we first searched for evidence of basal similarities among the orthologous promoters. In particular, we searched for the occurrence of TFBS spatial distribution patterns that are conserved in every member of each COG but not in paralogs [39]. We assumed that such patterns could provide a general measure of the degrees of conservation that occurred during the divergence of monocot and dicot lineages from their last common ancestor or during speciation of rice and sorghum.

**Figure 3:**
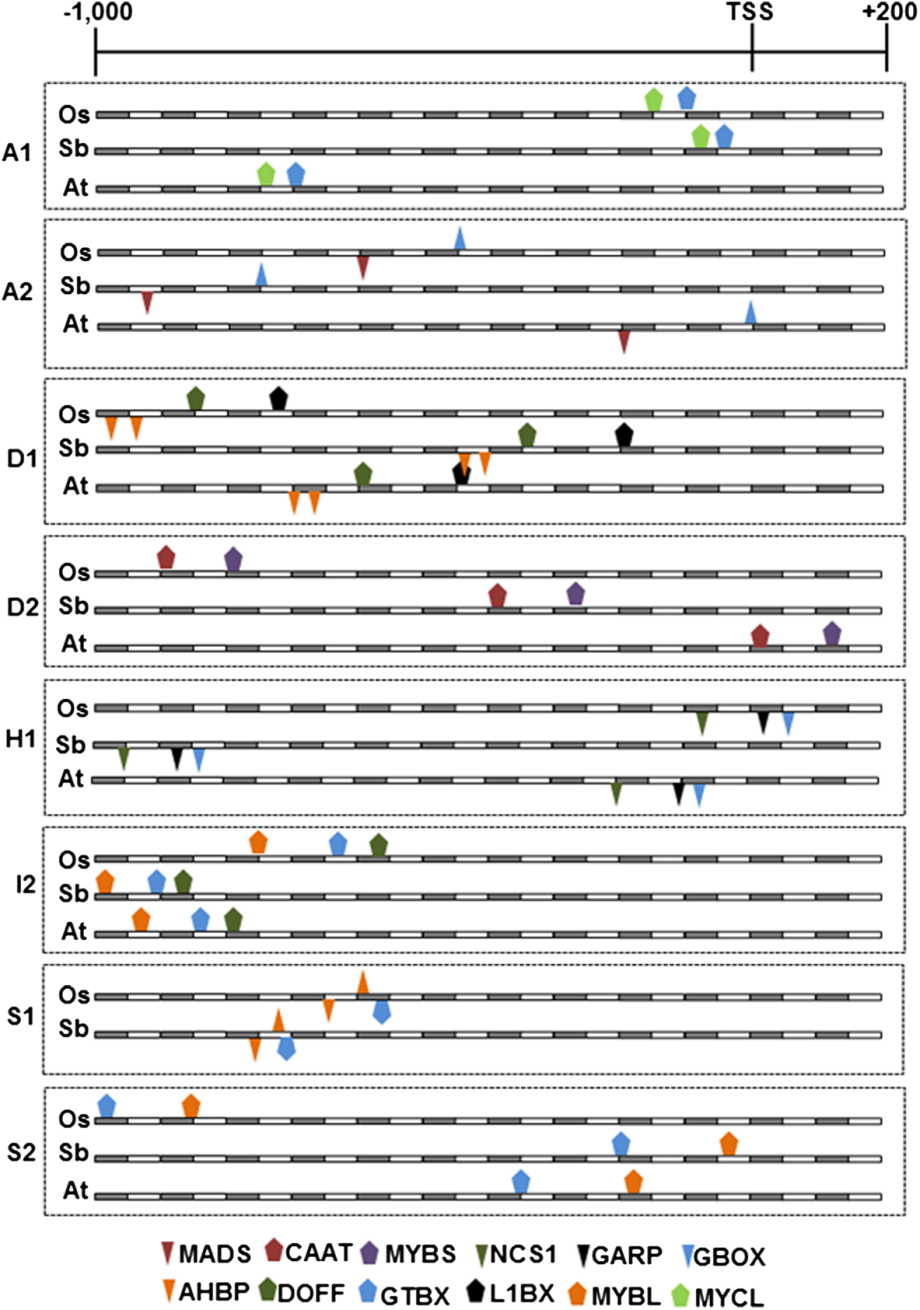
**Map and alignment of spatially conserved ‘core modules’ detected among the various COGs of *Oryza sativa *(Os), *Sorghum bicolor *(Sb) and *Arabidopsis thaliana *(At).** Inverted figures indicated the location of the TFBS sequence motif in different strands of the promoter region. TATA box is not shown in the maps but in most cases are located between 30 to 60 nt upstream from the TSS.

Conserved signatures occurring as modular combinations of two to four TFBS classes (referred to as ‘core modules’) were established in eight COGs (Figure 3). These core modules are comprised of either a combination of purely ortholog-specific TFBS classes or ortholog-specific plus ortholog/paralog-shared TFBS classes. Based on the occurrence and distribution of these modules, it was apparent that each tri-species COG was quite distinct even if they belong to the same functional sub-class of bZIP proteins. This variation suggests distinct signaling and regulatory mechanisms for each GOG, consistent with their presumed general biological function based on established classification [22].

The order, relative distance from each other and strand orientation of each putative TFBS that comprised a core module were remarkably conserved within each COG. Different patterns of relatedness were observed among rice, sorghum and Arabidopsis promoters in terms of the patterns of core module location relative to the TSS. Locational similarities between rice and sorghum core modules were more evident in A1, A2, D2 and S1. The other COGs showed either similar core module locations among all three species (I2) or similar locations between either of the monocot species and Arabidopsis (H1, S2) (Figure 3). Potential functional significance of the distance of the core modules from the TSS might be interpreted in terms of the efficiency by which these core modules interact with the core promoter during the formation of initiation complex. The spatially conserved core modules are comprised of various combinations of TFBS classes that were annotated in cis-element databases with key words associated with growth and developmental (MADS box, MYBS/MYB-R1, AHBP/HD-Zip, DOFF, GTBX/MYB, MYBL), gibberellic acid (GA) response (GARP/MYB-related), abiotic stress and pathogen defense (GBOX/bZIP, MYCL/bHLH, NCS1, CAAT/NF-y) related functions. The possible biological significance of these spatially conserved core modules may be interpreted in terms of the shared properties required for basal developmental regulatory programs of each orthologous promoter that were conserved during the divergence of monocot and dicot lineages from their last common ancestor or convergent parallel evolution of regulatory modules in the monocot and dicot lineages.

**Figure 4:**
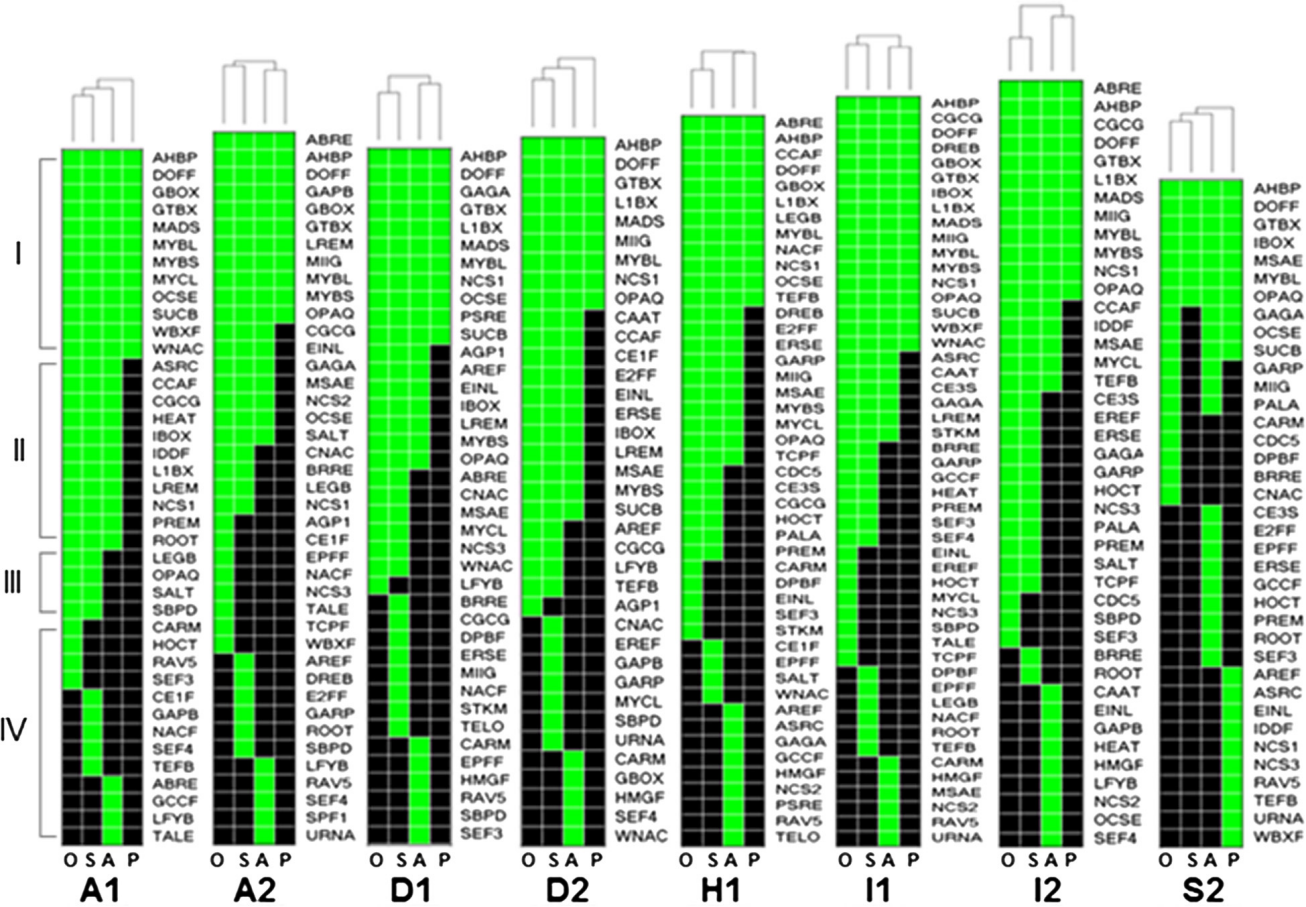
**Binary hierarchical dendograms showing four major patterns of clustering and relatedness among orthologous bZIP transcription factors from rice, sorghum and Arabidopsis based on cis-regulatory information content.** In all cases shown, rice (O) and sorghum (S) orthologs tend to cluster more closely with each other than with the Arabidopsis (A) orthologs and all other paralogs (P). Dendogram for COG A1 shows an example of the various patterns of TFBS co-occurrence that are also apparent in the other COGs. These patterns are defined by: TFBS classes that occurred/conserved in both orthologs and paralogs (**I**), TFBS classes present among all members of each tri-species COG but not in their paralogs, i.e., ortholog-specific (**II**), TFBS classes present in both monocot orthologs but not in the Arabidopsis ortholog, i.e., lineage-specific (**III**), and TFBS classes that are unique to each species, i.e., species-specific (**IV**). The occurrence of paralog-specific (P) TFBS classes was evident only in COG S2. Hierarchical clustering dendograms are presented only for representative COGs with a clear ortholog from all three species.

The occurrences of all TFBS classes included in this analysis are summarized in Additional file 3 and Additional file 4. Examination of the distribution of the total TFBS classes detected in each COG and those that were shared by COGs and paralogs revealed the large-scale similarities and differences in cis-regulatory information content as well as the organizational complexities of the promoters of stress-associated bZIP transcription factors. The distribution of TFBS classes in each COG clearly established four patterns of relatedness based on binary hierarchical clustering (Figure 4). The first pattern is characterized by the common occurrence of certain TFBS classes among orthologs and paralogs, reflecting the total regulatory information shared by all members of a given clade that was established by the radial tree (Figure 2).

The second pattern is defined by the occurrence of a given set of TFBS classes among the members of a given COG but not among paralogs (‘ortholog-specific’). The third pattern is defined by the common occurrence of a given set of TFBS classes among the orthologous monocot genes but not in the orthologous dicot gene(s) and paralogs (‘lineage-specific’). The fourth pattern is defined by certain TFBS classes with unique occurrence in either rice, sorghum or Arabidopsis orthologs, reflecting the differences that occurred during speciation (‘species-specific’). No robust trend was detected among the paralogs to indicate a clear pattern of ‘paralog-associated’ TFBS occurrence except in S2, consistent with the presumed diverse functions of paralogs.

The binary hierarchical clustering dendograms in Figure 4 also further revealed the tendency for the monocot species to be more closely related to each other than to the dicot Arabidopsis in terms of cis-regulatory information content, mirroring the exact same trend established by the coding sequence (nucleotide and amino acid levels) phylogeny. This general trend could also be seen (at least slightly) from the patterns shown in Table 1 and Figure 3. All these results are consistent with the basic assumptions of phylogenetic footprinting and imply that the patterns of upstream sequence similarities and differences established for this small group of bZIP transcription factors are robust enough to be used for meaningful functional and biological inferences [27,40,41].

### Functional implications of ortholog-specific signatures

The TFBS classes shared in common by the stress-associated orthologs with other paralogous genes are largely associated with basal growth and developmental functions, ranging in occurrence from a simpler non-modular combinations of five TFBS classes per cluster to a more complex but non-modular combinations of more than ten TFBS classes per cluster (Figure 4, Additional file 5). More than 75% of the shared TFBS classes have been annotated in cis-element databases with keywords related to either vegetative or reproductive developmental processes. The DOFF/Zinc finger, MYBL, AHBP/HD-zip, L1BX/homeodomain, GTBX, NCS1 elements have the broadest occurrences across the clusters. Interestingly, many of these TFBS classes are also among the components of the spatially conserved core modules shown in Figure 3. However, the combinatorial (or modular) occurrence of these elements with other elements and their spatial distribution are not conserved among paralogs in the same way that they are conserved as modules in the COGs. Based on these trends, it appears that the functionality of these elements in terms of stress regulation of orthologs is defined by their modular nature and such organization are apparently lost in the paralogs [41,42].

Ortholog-specific signatures were also evident from the results of hierarchical clustering analysis, reflecting the additional non-species-specific or non-lineage-specific regulatory information that may be required for the stress-associated function of the orthologs in conjunction with the spatially conserved core modules (Figure 4, Additional file 6). Of the total 18 TFBS classes that occurred in an ortholog-specific manner, about 67% (12) have been annotated in databases with key words related to abiotic and biotic stress responses including temperature extremes, dehydration, UV, oxidative stress, salinity, pathogens and wounding as well as responses related to ABA and ethylene signaling. The other TFBS classes (33%) that were detected only among the orthologs but not in paralogs are associated with various growth and developmental processes including the regulation of cell cycle, morphogenesis and circadian responses. These trends reiterate that the subset of ortholog-specific TFBS classes that probably work in conjunction with the spatially conserved core modules are defined primarily by putative stress-related regulatory functions.

The binary hierarchical clustering dendogram also shows that each COG is unique by virtue of their cis-regulatory information signatures (Figure 4, Additional file 6). Even the COGs that belong to the same functional class (for example, A1/A2 and D1/D2) have very distinctive patterns, distinct enough to place them in distant phylogenetic branches. The biological significance of these elements may be interpreted in terms of their possible roles as ‘regulatory fine-tuners’, perhaps by facilitating the integration of various types of intrinsic (growth) and extrinsic (stress) signals that contribute to the overall expression potential of the gene.

In general, the functional annotations of the TFBS classes that characterized a given COG reflect the functional sub-class of bZIP proteins to which the specific COG belongs. For instance, A1 and A2 belong to the sub-class involved in ABA-mediated stress response signaling (Figure 2). Both COGs contain at least one class of ABA-associated TFBS such as IBOX/Zinc finger for A1 and AGP1/GATA, OPAQ/bZIP, CE1F, and IBOX/Zinc finger for A2, with IBOX/Zinc finger as the core element shared by both A1 and A2 (Additional file 6). Hierarchical clustering dendograms also showed the expected patterns of co-occurrence between TFBS classes associated with specific signals. For instance, co-occurrence among ABA-related TFBS classes is quite apparent in most cases as well as the co-occurrence of TFBS classes involved with mechanisms associated with oxidative and pathogen defenses.

Another apparent positive association is shown by D1 and D2, which belong to the sub-class involved in pathogen and oxidative stress associated bZIP proteins (Figure 2). The cis-regulatory signatures of these COGs overlap through the EINL and LREM elements, both of which are associated with AP2/ERF-type transcription factors involved in disease response mechanisms via oxidative and ethylene signal transduction pathways (Additional file 6). However, D1 and D2 are also unique by virtue of the occurrence of other oxidative-associated elements such as AGP1/GATA in D1 and ERSE/bZIP and CAAT/NF-y in D2. In addition to the salient regulatory features of D1 and D2 that were consistent with their presumed roles in oxidative and pathogen defense mechanisms, each COG also contains distinct sets of other elements involved in ABA response mechanism, suggestive of how the regulation of the members of this COG might involve the interplay between oxidative, ethylene and ABA signal transduction pathways [29,43]. Of all the COGs, D2 also has the most complex combination of stress-associated TFBS based on the highest density in all its members. This trend appears to be consistent with the presumed complex roles of D2 members in both abiotic and biotic (disease and wounding) stress response mechanisms [22,24].

### Lineage-specific signatures

**Figure 5:**
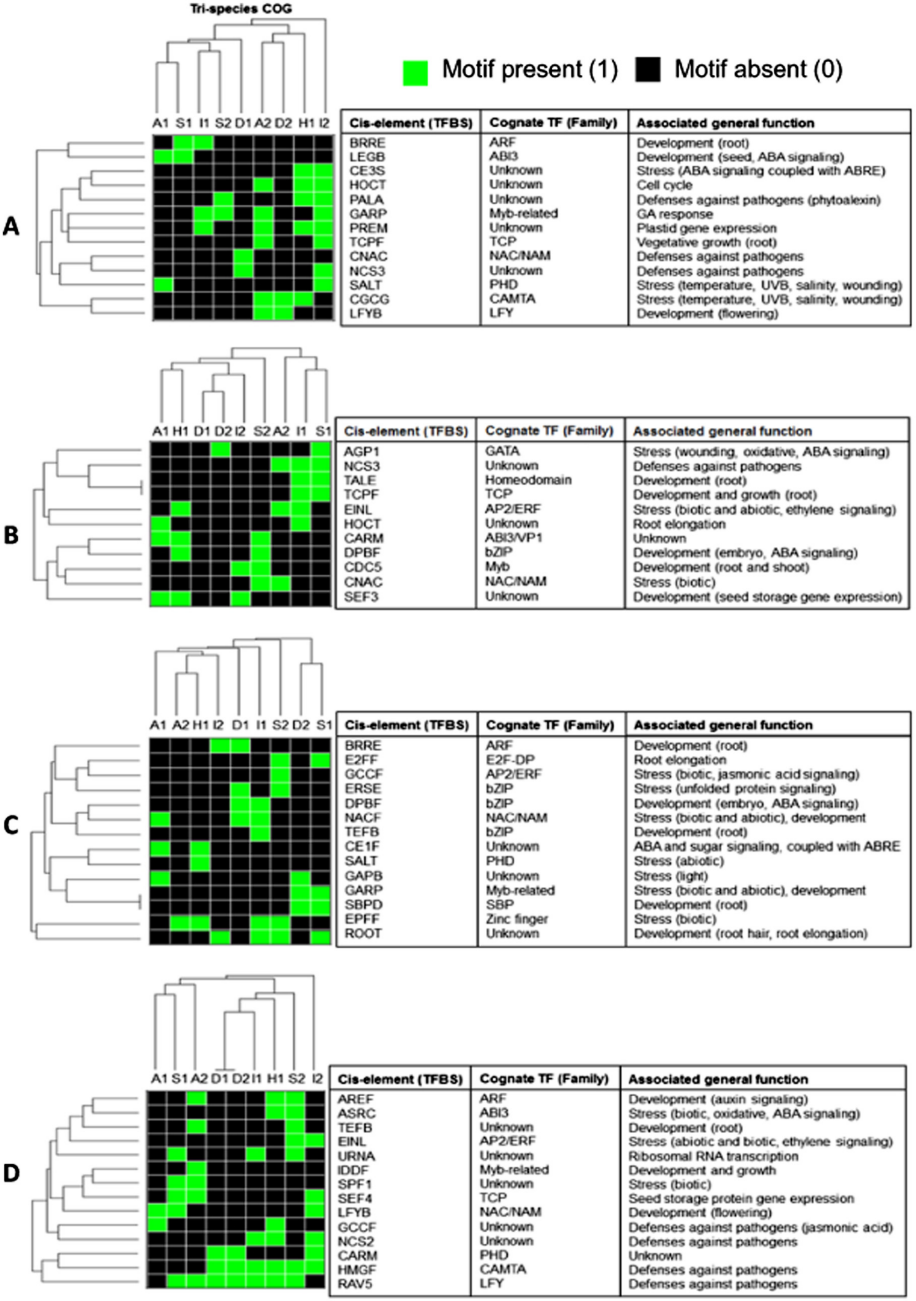
**Binary hierarchical dendograms showing the clustering patterns of: (A) TFBS classes that are found in the two monocot (rice, sorghum) members of a given COG but not in the dicot (Arabidopsis) member, and also not among paralogs (lineage-specific), (B) TFBS classes occurring uniquely among rice orthologs (species-specific), (C) TFBS classes occurring uniquely among sorghum orthologs (species-specific), and (D) TFBS classes occurring uniquely among Arabidopsis orthologs (species-specific).** The associated transcriptional regulators (TF) and their known biological functions based on the annotation in the plant-specific cis-element (Genomatix, TRANSFAC and PLACE) databases are shown for each TFBS class.

Each COG was found to contain a number of putative TFBS classes that were shared by the two monocot species but absent in the orthologous genes from Arabidopsis and vice versa (Figure 4). This type of variation may be interpreted as a possible indication of distinct stress-regulatory components that evolved after the divergence of the monocot and dicot lineages from their last common ancestor. The subset of putative TFBS classes specific to the monocot members in each COG are equally represented by both stress-associated (46%) and development-associated (46%) elements (Figure 5A). The development-associated, monocot-specific TFBS classes are primarily involved in root, seed, and vegetative organ development (BRRE/ARF, LEGB/ABI3, TCPF) and GA-response (GARP/Myb-related). The stress-associated TFBS classes include several that are involved in ABA signaling (LEGB/ABI3, CE3S) and other types of abiotic stress factors such as temperature extremes, UV, salinity and wounding (CGCG/CAMTA). Abundance of elements associated with the regulation of plant responses to pathogens such as PALA, CNAC/NAC/NAM, NCS3 and SALT/PHD are quite apparent.

Similar trends were observed in the Arabidopsis-specific TFBS classes, where both development-associated (29%; AREF/ARF, SEF4/TCP) and stress-associated TFBS classes were represented (Figure 5D). As in the monocots, the stress-associated TFBS classes that are specific to Arabidopsis orthologs were also characterized by functions associated with the regulation of defenses against pathogens (43%), including those involved in oxidative, ethylene, and jasmonic acid (JA) signaling (ASRC/ABI3, EINL/AP2/ERF, GCCF, HMGF/CAMTA, RAV5/LFY) [44]. Based on these trends, it appears that the major difference in cis-regulatory information content between the monocot and dicot lineages could be defined in terms of the types of elements with likely important roles in disease-regulated expression. This trend seems to support the possibility that distinct mechanisms are involved in the regulation of orthologous stress-related bZIP transcription factors in monocot and dicot species, conferred by the regulatory fine-tuners interacting with the spatially conserved core modules, perhaps because of the distinct sets of pathogens that co-evolved with monocot and dicot plants.

### Species-specific signatures

Rice and sorghum are related members of the grass (Poaceae) family representing about 70 million years of evolutionary history. We compared the various orthologous pairs in rice and sorghum for nine of the ten COGs shown in Figure 2, in order to establish patterns that could provide a glimpse of the finer-scale changes in cis-regulatory complexity as a result of speciation. Like in the other levels of comparison on ortholog vs. paralog and monocot ortholog vs. dicot ortholog, rice vs. sorghum comparison also revealed distinct cis-regulatory signatures defined by various combinations of stress-associated (occurrence of 36% in rice and 57% in sorghum) and growth and development-associated (occurrence of 55% in rice and 42% in sorghum) TFBS classes (Figure 5B and 5C). However, the most apparent commonality between rice and sorghum orthologs, which also distinguished them from Arabidopsis orthologs was the pronounced abundance of TFBS classes associated with the regulation of root development and growth, with occurrences of 36% in rice and 36% in sorghum [45]. It is possible that the specification of root expression in rice and sorghum are facilitated by distinct combinations of root-specific regulatory signals, with the rice orthologs making use of TALE/Homeodomain, TCPF, HOCT and CDC5/MYB elements, and sorghum orthologs making use of BRRE, E2FF/E2F-DP, SBPD and ROOT elements. This trend was not particularly conserved among Arabidopsis orthologs for most of the COGs, where only one class of putative root-associated TFBS (TEFB) was detected (Figure 5D).

Root function plays an important role in stress responses, particularly in relation to dehydration and osmotic stresses. The trends established based on our current results may then be interpreted in terms of possible differences in the regulation of stress response transcriptomes in the roots of rice and sorghum. It is interesting to note that rice is a C_3_ plant that thrives best under semi-flooded conditions, hence very sensitive to even mild dehydration, while sorghum is a C_4_ plant that exhibits high level of tolerance to drought. Differences in the configurations of the root transcriptomes might be a possible implication of these patterns, which may also be relevant to the fundamental differences in root development and physiology between rice and sorghum.

### Integration of developmental and stress-related responses

**Figure 6:**
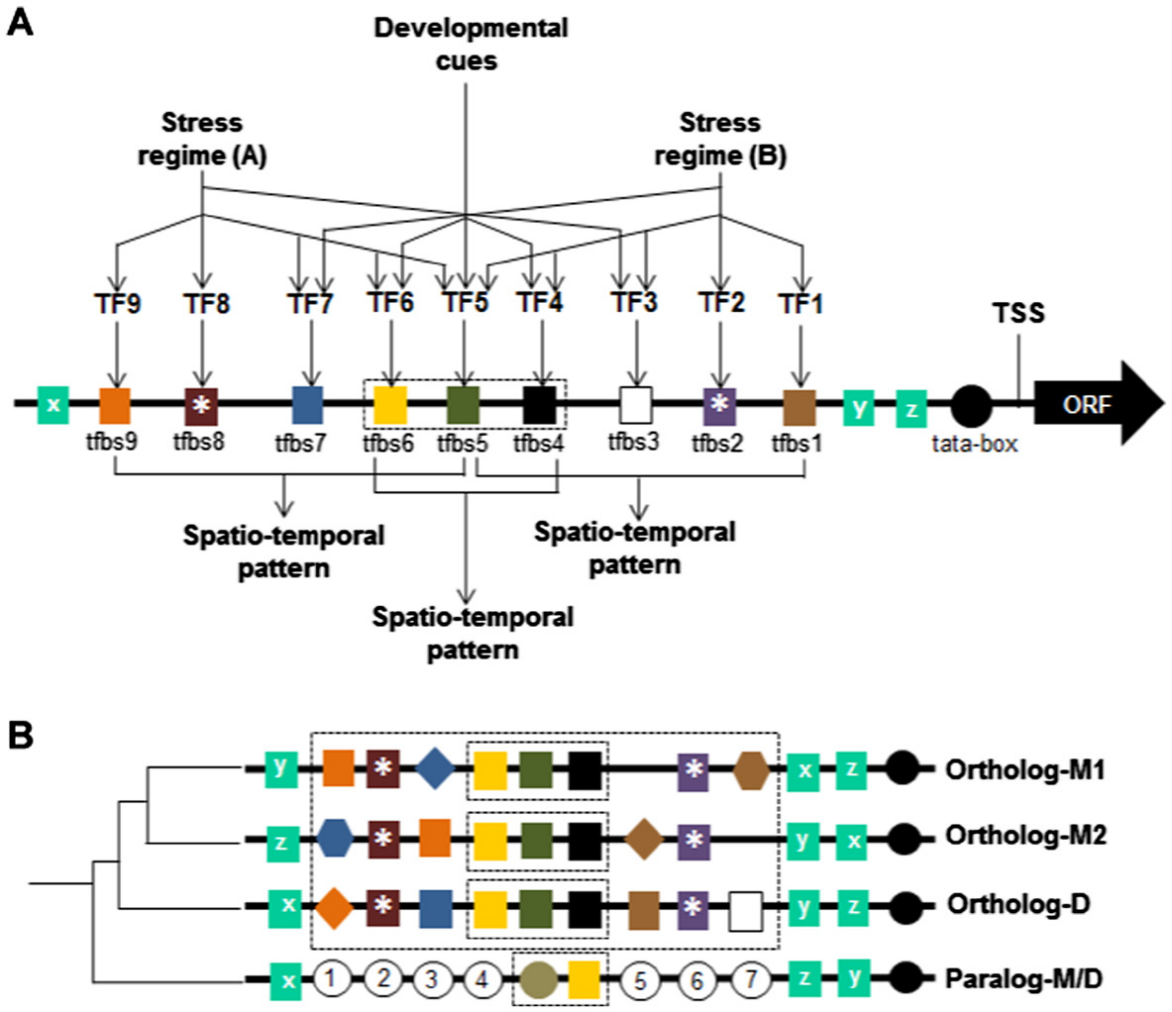
**Hypothetical models explaining the potential biological significance of the cis-regulatory patterns revealed by phylogenetic footprinting.** (**A**) Simplistic model of a hypothetical promoter from a stress-regulated bZIP transcription factor. Each colored box (tfbs1, tfbs2, etc.) represents a putative TFBS class recognized by specific cognate DNA-binding proteins (TF1, TF2, etc.). The boxes corresponding to tfbs-x, tfbs-y, and tfbs-z represent elements that are conserved between stress-associated orthologs and non-stress-associated paralogs. The spatially conserved ‘core module’ (boxed) is found in all orthologs but not in paralogs and is likely to define the basal regulatory program of the gene. The other tfbs classes outside the ‘core module’ represent the ‘regulatory fine-tuners’ that tend to exhibit lineage or species specificity. The tfbs classes marked with an asterisk represent the ‘regulatory fine-tuner’ that is likely specific and/or critical for a given stress signal. The model postulates that distinct signals act on a combination of tfbs, thus responses are configured by synergistic interaction of several TFs. The tfbs-TF combinations overlap between different stress and developmental signals facilitating an integrated response and distinct spatio-temporal pattern for each member of an orthologous group. (**B**) Simplistic model showing the divergence of cis-regulatory information content among orthologs and paralogs. The spatially conserved ‘core module’ (boxed) is conserved among the orthologs from monocot species (M1 and M2) as well as in the ortholog from the dicot species (D). The ‘core module’ diverged between orthologs and paralogs. In addition, both monocot (M) and dicot (D) paralogs have acquired other elements, represented by numbered white circles.

A given gene may be expressed through multiple independent or convergent signaling pathways involving different combinations of regulators. The overall genetic machinery that allows certain genes to be expressed in response to certain physical or chemical stress signals is therefore tightly integrated with basal growth and developmental programs. As illustrated in the hypothetical model established according to the patterns of cis-regulatory conservation (Figure 6A), the overall expression potential of a given gene is defined by synergistic interaction of various elements and their cognate regulator proteins. This synergy may be critical to the finer-scale spatio-temporal differences of genes that perform homologous biological functions across species with distinct ecological adaptation such as rice, sorghum and Arabidopsis, at least according to the trends revealed by the gene subset examined in this study.

At the very heart of the regulatory sequence conservation among orthologous bZIP transcription factors are the spatially conserved core modules, which appear to be the primary determinants of the basal developmental and spatial regulatory programming. The function of a core module is likely to be further elaborated or specified in relation to specific growth response and stress signals by interaction with the regulatory fine-tuners and their cognate regulator proteins [46]. Core modules tend to be highly conserved during speciation while the regulatory fine-tuners appear to have greater evolutionary flexibility leading to both lineage-specific and species-specific signatures (Figure 6B).

Overall, the resolution afforded by the analysis of tri-species COGs revealed a general trend in which orthologous stress-associated bZIP transcription factors of rice, sorghum and Arabidopsis have finer-scale differences in regulatory information content, both qualitatively and quantitatively, despite the presumed conservation of broad biological function (i.e., stress response). Certain orthologs appeared to have either lost or gained specific regulatory information, and these may have important roles in defining their unique spatio-temporal expression profiles in response to complex combinations of extrinsic and intrinsic signals. These differences can be viewed as possible indications of how natural selection may have impacted the divergence of this group of stress-related transcription factors presumably from their ancestral role of being involved primarily in regulating growth and development [1]. Possible ways that this may have occurred are by the acquisition of additional regulatory signals or maintenance of ancestral regulatory signals that have been favored by natural selection.

## Conclusions

Phylogenetic footprinting is based on the overall assumption that upstream sequence motifs that are highly conserved between homologous genes represent functional regulatory elements [27,28,39,41]. Conventional use of this paradigm has focused primarily on the identification of highly conserved elements that define the narrow-scale expression similarities between homologous genes in response to specific signals. The inherent limitation of this relatively simplistic assumption is that it tends to neglect an important view that homologous genes may exhibit expression similarities in response to certain signals and still be unique in a regulatory context if the totality of cis-regulatory information content that defines the large-scale or finer-scale spatio-temporal programming details is taken into consideration. For instance, homologous stress-related genes from two divergent species (orthologs) may share a common cis-element that is critical to stress inducible expression. However, it is also quite conceivable that the regulatory mechanism that integrates stress-related signals with growth and development and with other intrinsic and extrinsic signals that define the gene’s overall expression potential would vary between two evolutionarily diverse species (Figure 6A).

This study is an attempt to extrapolate on the basic assumptions of phylogenetic footprinting to allow us to dissect the finer details that contribute to the distinct cis-regulatory signatures of orthologous stress-related bZIP transcription factors from rice, sorghum and Arabidopsis beyond the more obvious patterns of conservation. The patterns and relationships that we have revealed appear to be robust and quite meaningful for use as conceptual basis of further empirical validation. Furthermore, the trends that we have established have potential physiological significance given that the comparison involved three species with distinct stress physiological properties and adaptation regimes. Our comparisons provided meaningful contrasts at various levels by representing monocot vs. dicot promoter structures, syntenic (rice vs. sorghum) and non-syntenic (rice/sorghum vs. Arabidopsis) genomes, and a spectrum of natural variation for relative sensitivity to various forms of abiotic stresses. Rice is a C_3_ Gramenae that can withstand only periodic and very mild cold stress and thrives best in semi-flooded soil, while sorghum is a cold-sensitive but drought-tolerant C_4_ Saccharinae and Arabidopsis is a temperate C_3_ Brassicaceae that acquires freezing tolerance by cold acclimation [33,47,48].

Indeed, the patterns of cis-regulatory conservation revealed in this study were consistent with the established functional sub-classification of bZIP transcription factors, illustrating the strength of the paradigm. For instance, orthologous genes that belong to Group-A (A1, A2) involved in ABA-mediated stress signaling exhibited the characteristic ABA response-associated cis-elements at various combinatorial complexities. Likewise, orthologous genes that belong to Group-D (D1, D2) involved in pathogen and oxidative defenses exhibited the characteristic cis-regulatory signatures expected of such mechanisms [22,24].

Subtle differences in regulatory information content among orthologous genes were established even at the species level. These subtleties suggest that each member of an orthologous group is potentially unique within the context of regulation. The uniqueness of each member could be a reflection of additional functional attributes that may confer regulatory specificity or precision. Potential biological implications include possible effects of changes in transcription factor regulation to network rewiring and its consequence to the evolution of transcriptional and biochemical network complexities.

Whether the relationships revealed in this relatively small window of information reflect the overall global trends remains to be seen with the analysis of a larger set of orthologous groups from diverse plant species. With the rapid progress in sequencing and annotating representative genomes from virtually any group of flowering plants, the patterns revealed in this study can be validated using a more encompassing number of reference genomes representing a more closely spaced evolutionary continuum. Moreover, the validity of our biological interpretations of the conservation patterns requires fine-scale spatio-temporal expression matrix across a battery of stress conditions throughout the plant’s entire life cycle, which is now achievable by comparative deep sequencing of transcriptomes. Finally, the robust phylogenetic footprints established based on this strategy revealed different patterns of finer-scale regulatory sequence signatures allowing a different layer of contrast between species and perhaps a new perspective that may be useful in understanding the contribution of promoter restructuring to the diversity of stress network complexities in flowering plants.

## Methods

### Validation of rice bZIP transcription factor expression

The composition of the core subset of stress-associated bZIP transcription factors of rice included in this study was based on the low temperature and H_2_O_2_ (4 mM) response transcriptomes described previously [23,26]. Additional expression studies on the 11 genes included in the core subset in response to rapid dehydration and high salt concentration was performed by qRT-PCR following the established procedures [3,26]. Briefly, three to four leaf stage (V_3_ to V_4_) seedlings of japonica rice cultivar Nipponbare were first established in standard Yoshida hydroponic medium (12 hour photoperiod, 29 °C/24 °C temperature regime) for four days prior to stress treatments. Seedlings were subjected to salinity stress by transferring from Yoshida medium to an aqueous solution of 300 mM NaCl. Rapid dehydration was imposed by complete withdrawal from the hydroponic tubs. Seedlings were maintained in a growth chamber at 28 °C constant temperature with 50% relative humidity during the entire period of rapid dehydration. Transcript abundance was expressed as normalized values against a constitutively expressed actin gene and relative to the control values. Hierarchical clustering of relative expression was performed with the TMEV analysis suite [49].

### Phylogenetic reconstruction of orthologs and paralogs

Genomic loci corresponding to the best hits of each rice gene were identified in sorghum and Arabidopsis by BlastX and BlastN searches of the respective reference genome sequences with the rice full-length CDS as query [33,50]. Parallel BlastX and BlastN analysis yielded identical hits with the output of BlastP searches. Results were further examined by comparing the sequence alignments of the bait rice gene with the primary hits (putative orthologs) and secondary hits (putative paralogs) from each species for each functional sub-class of bZIP transcription factors through the ClustalW version 1.83 [51].

In order to establish the various clusters of orthologous groups (COGs) in relation to the accepted functional classification scheme of bZIP transcription factors [22,24], the full-length CDS of putative orthologs and paralogs identified were used for phylogenetic reconstruction by Neighbor-Joining (NJ) method, implemented in the Molecular Evolutionary Genetic Analysis (MEGA) version 3.1 [52]. Analysis was conducted with complete deletion option for gaps and missing data and with the Poisson correction model for distance computation. Bootstrap analysis with 1,000 replicates was conducted to examine the statistical reliability of the tree topology and nodes with a bootstrap cut-off score of 50%. Orthologous and paralogous relationships established through this method were further verified for consistency with the public comparative genome annotation [35].

### Detection and identification of TFBS motifs

Upstream sequences (−1,000 to +200 relative to transcription start site or TSS) of the orthologous genes and their representative paralogs were extracted from reference genome sequences through the Gramene comparative genomics browser [35]. In rice and Arabidopsis, TSS was established either by *ab initio* prediction or through the *bona fide* TSS revealed by the alignment of full-length cDNAs or FL-EST contigs with the reference genomes [50,53-55]. Delineation of the −1,000 to +200 regions in sorghum orthologs and paralogs were based on the *ab initio* predicted TSS [35]. The 1,200 bp upstream sequences of orthologs and paralogs were scanned for statistically significant occurrence of sequence motifs (6 to 8 nucleotides) representing potential transcription factor binding sites (TFBS) or cis-elements, primarily using the Genomatix Suite [56]. The output was matched with the most likely TFBS using the MatInspector program Release 7.7.3.1 comprised of a large library and matrix description of known plant-specific cis-elements and their cognate regulators. The parameters used were the standard core similarity and optimized matrix similarity. Independent validation of the TFBS classes detected by Genomatix for D1 and D2 was conducted by parallel analysis with the Dragon Motif Builder algorithm with EM2 option and 0.875 threshold and identification by matching with entries at the PLACE and TRANSFAC databases [26,57-59]. TFBS classes established in parallel by the two methods were nearly identical. Relative TFBS enrichment was compared between species for each COG by chi-square analysis of species-to-species ratios. TFBS occurrences were expressed in binary format (1 = single copy to multi-copy occurrences, 0 = absent) and the data matrices were hierarchically clustered to search for patterns of similarities between species, patterns of co-occurrence between TFBS classes and patterns of similarities between the various COGs. Hierarchical clustering was performed using the TMEV analysis suite [49].

## Abbreviations

COG: Cluster of orthologous groups; TFBS: Transcription factor binding site; bZIP: basic leucine zipper; ABA: Abscisic acid; GA: Gibberellic acid; JA: Jasmonic acid; CDS: Coding sequence; TSS: Transcription start site; FL-EST: Full-length expressed sequence tags; qRT-PCR: quantitative real-time polymerase chain reaction.

## Competing interests

The authors declare no competing interest in connection to the work described in this report.

## Authors’ contributions

FX performed the gene orthology analysis, phylogenetic reconstructions, TFBS detection and phylogenetic footprinting by Genomatix, and all statistical analyses. MRP, VH, AK and SJY contributed to the transcriptome analysis and additional profiling studies by real-time PCR. BM performed motif analysis by the Dragon Motif Builder. BGDR was responsible for the overall concept and experimental designs, data integration, analysis and interpretation, and wrote the entire manuscript. All authors approved the final manuscript.

## Supplementary Material

Additional file 1 Phylogenetic reconstruction of orthologous and paralogous bZIP transcription factors in *Oryza sativa* (Os), *Sorghum bicolor* (Sb) and *Arabidopsis thaliana* (At). Click here for file

Additional file 2 **List of total TFBS classes detected among orthologs and paralogs through the Genomatix promoter detection algorithm.** Annotation is based on Genomatix and TRANSFAC databases. Click here for file

Additional file 3 Frequency of occurrence of the ortholog-specific TFBS classes in different COGs. Click here for file

Additional file 4 Frequency of occurrence of the ortholog-specific TFBS classes in different COGs. Click here for file

Additional file 5 **Binary hierarchical dendogram showing the clustering patterns for TFBS classes that are common between the stress-associated orthologs and their non-stress-associated paralogs.** The associated transcriptional regulators (TF) and their known biological functions based on the annotation in the plant-specific cis-element (Genomatix, TRANSFAC and PLACE) databases are shown for each TFBS class. Click here for file

Additional file 6 **Binary hierarchical dendogram showing the clustering patterns of ortholog-specific TFBS classes.** TFBS classes shown are found among the members of the tri-species COGs but not among their paralogs. The associated transcriptional regulators (TF) and their known biological functions based on the annotation in the plant-specific cis-element (Genomatix, TRANSFAC and PLACE) databases are shown for each TFBS class. Click here for file
