# Supplementary figures and images for: Cis-regulatory signatures of orthologous stress-associated bZIP transcription factors from rice, sorghum and Arabidopsis based on phylogenetic footprints
Source: BMC Genomics. 2012 Sep 20;13:497. doi: 10.1186/1471-2164-13-497 (PMC3522565; doi:10.1186/1471-2164-13-497)

## Slide 1
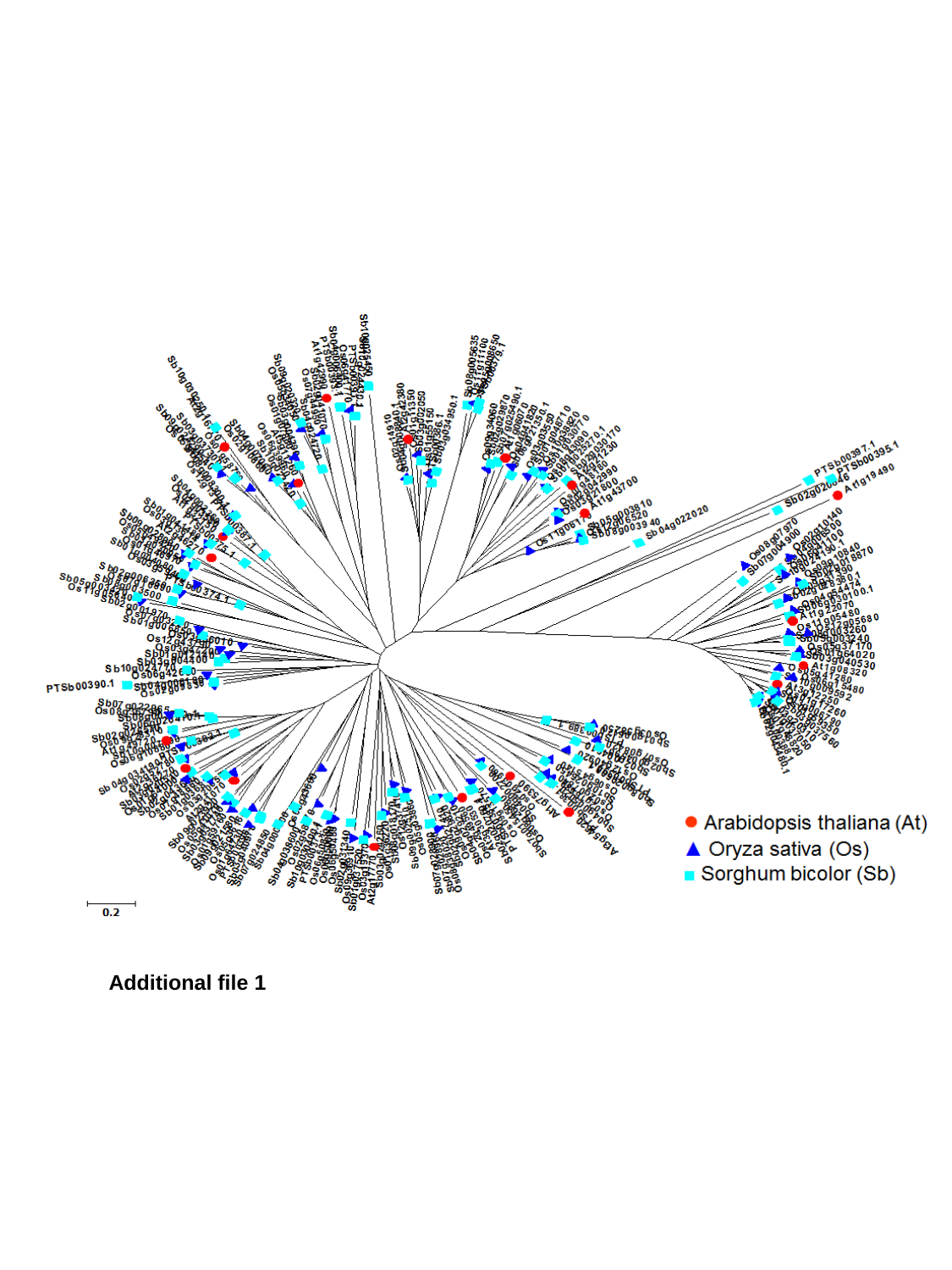

Additional file 1

Supplement: Additional file 1 — Phylogenetic reconstruction of orthologous and paralogous bZIP transcription factors in Oryza sativa (Os), Sorghum bicolor (Sb) and Arabidopsis thaliana (At). [file 1471-2164-13-497-S1.pptx]

## Slide 1
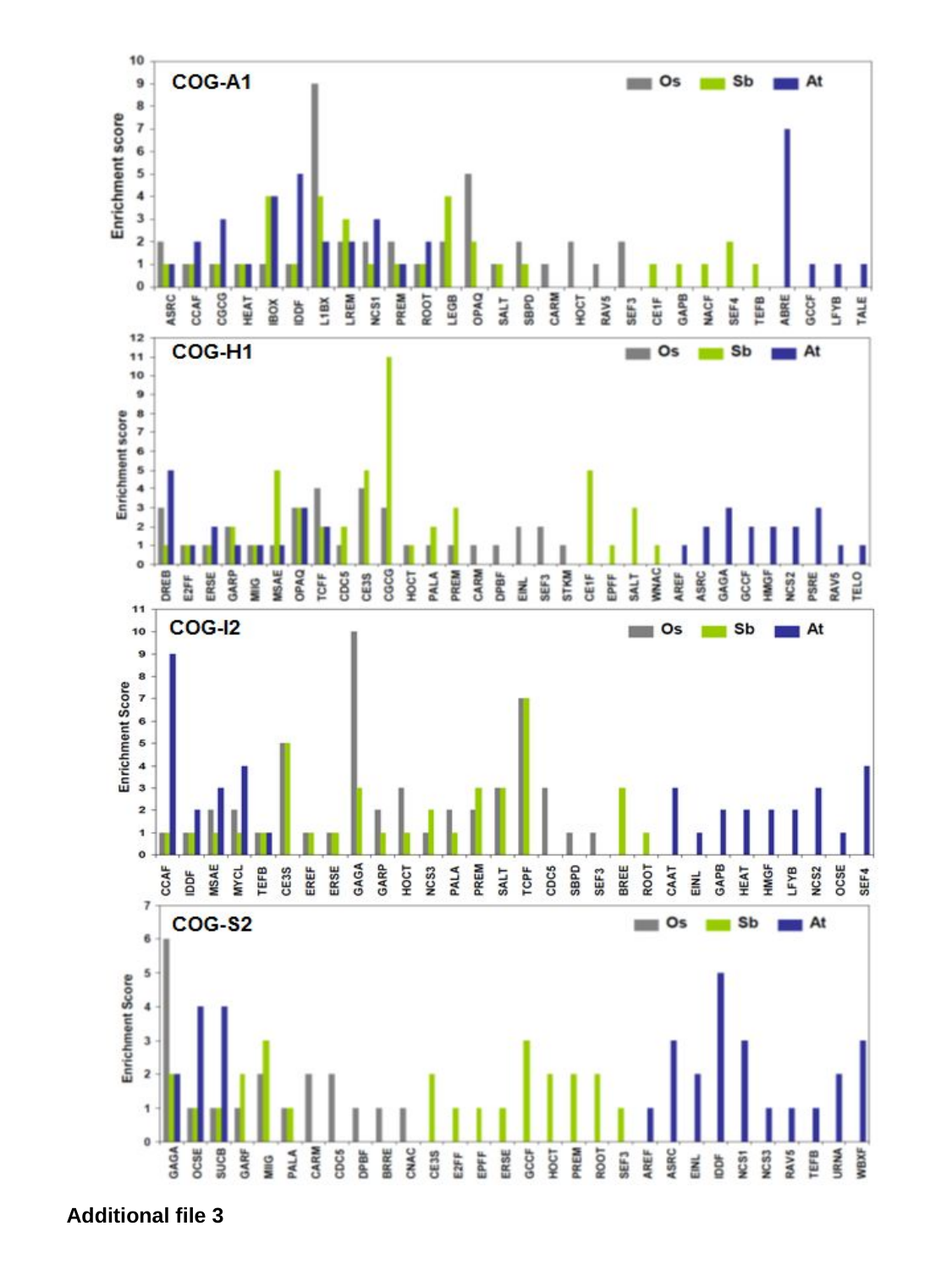

Additional file 3

Supplement: Additional file 3 — Frequency of occurrence of the ortholog-specific TFBS classes in different COGs. [file 1471-2164-13-497-S3.pptx]

## Slide 1
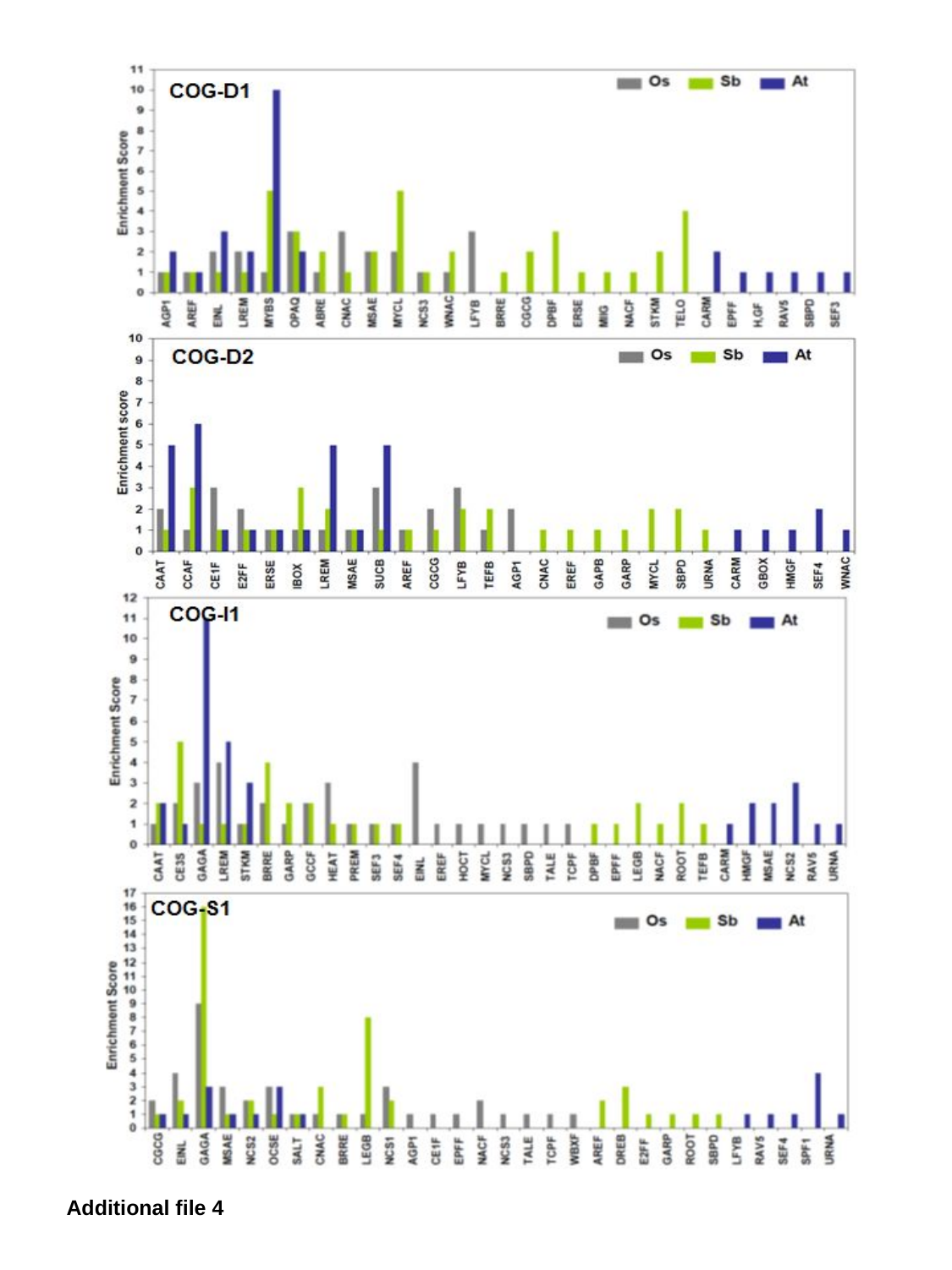

Additional file 4

Supplement: Additional file 4 — Frequency of occurrence of the ortholog-specific TFBS classes in different COGs. [file 1471-2164-13-497-S4.pptx]

## Slide 1
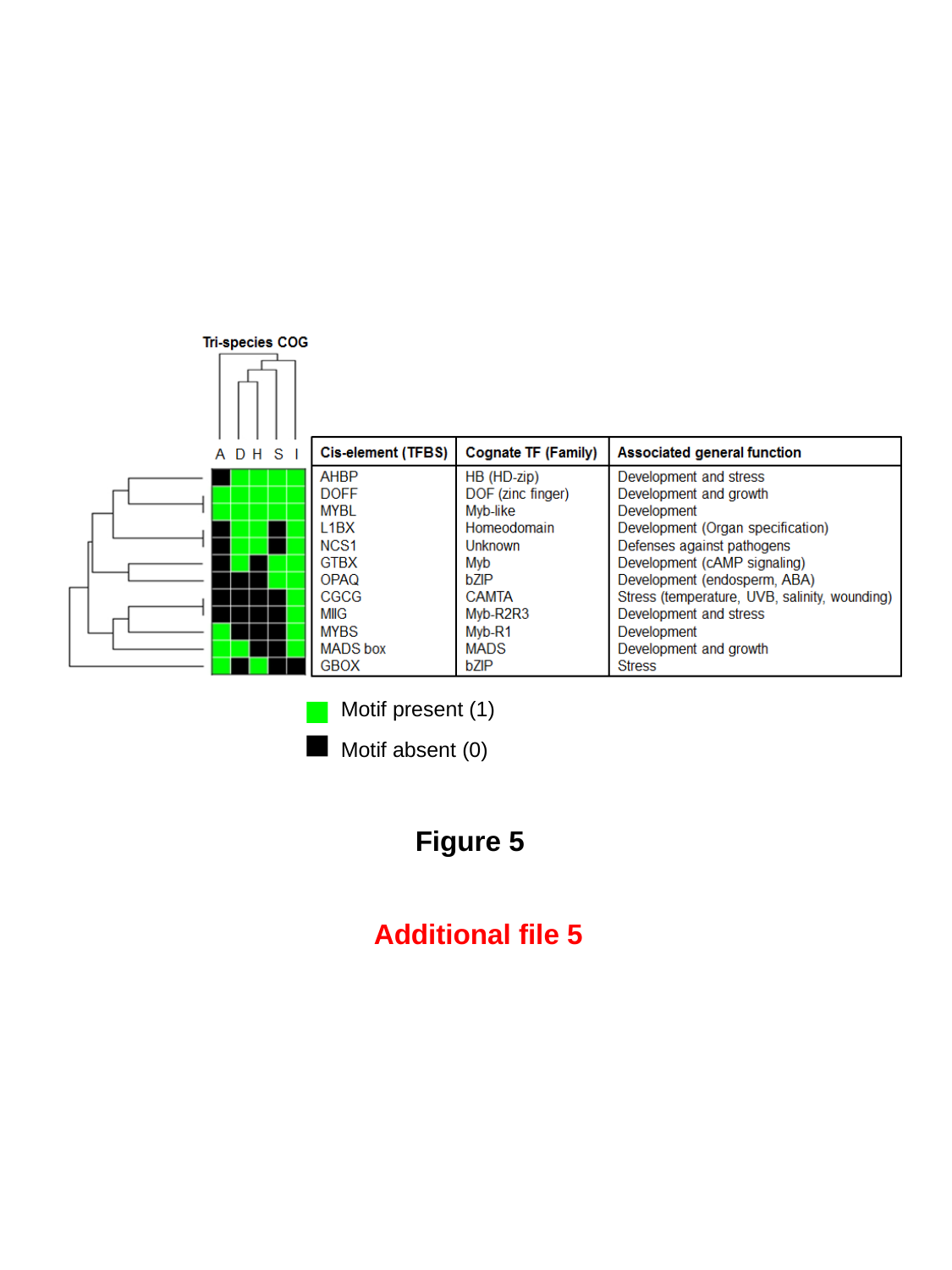

Motif present (1)
Motif absent (0)
Figure 5
Additional file 5

Supplement: Additional file 5 — Binary hierarchical dendogram showing the clustering patterns for TFBS classes that are common between the stress-associated orthologs and their non-stress-associated paralogs. The associated transcriptional regulators (TF) and their known biological functions based on the annotation in the plant-specific cis-element (Genomatix, TRANSFAC and PLACE) databases are shown for each TFBS class. [file 1471-2164-13-497-S5.pptx]

## Slide 1
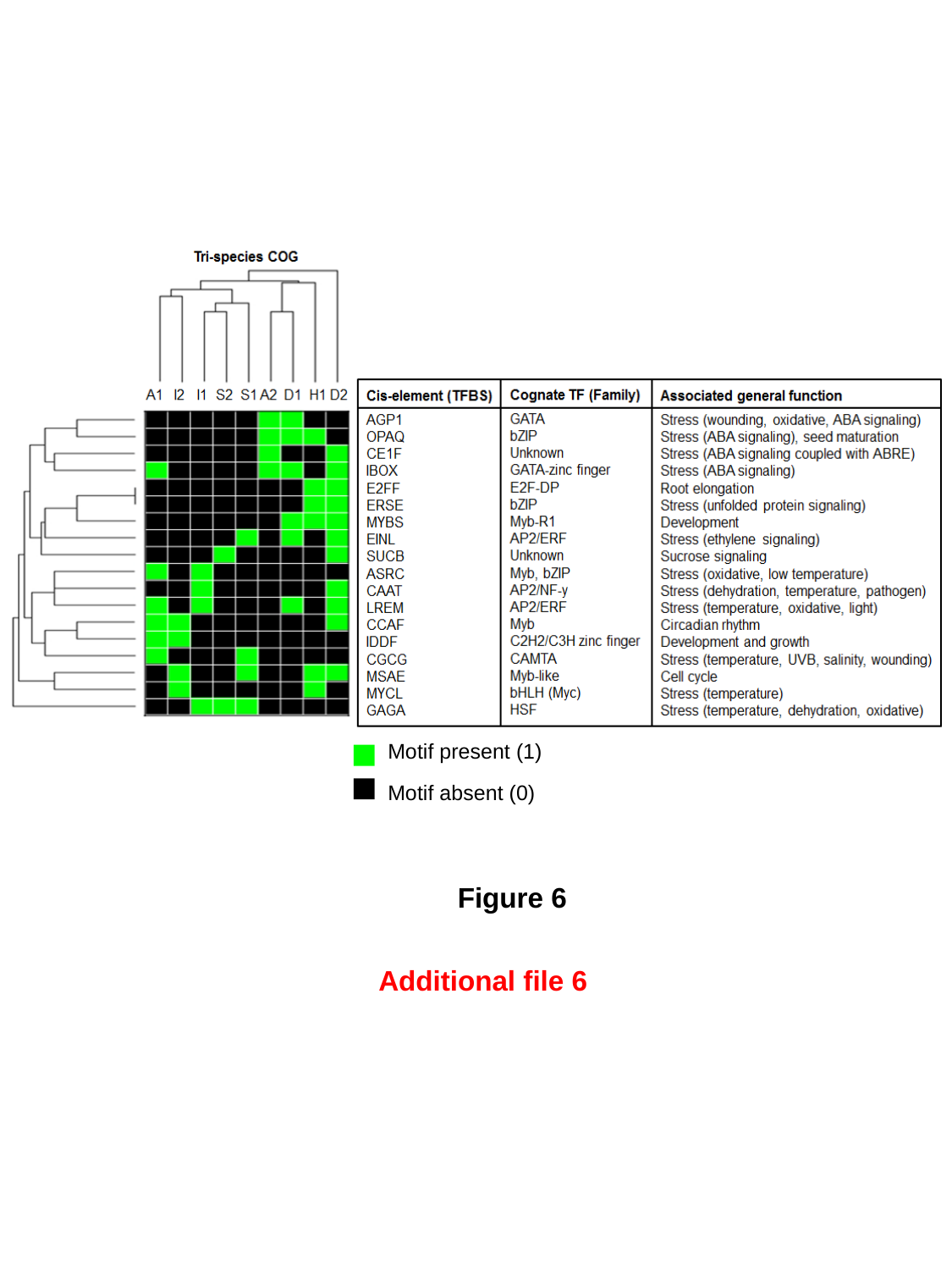

Motif present (1)
Motif absent (0)
Figure 6
Additional file 6

Supplement: Additional file 6 — Binary hierarchical dendogram showing the clustering patterns of ortholog-specific TFBS classes. TFBS classes shown are found among the members of the tri-species COGs but not among their paralogs. The associated transcriptional regulators (TF) and their known biological functions based on the annotation in the plant-specific cis-element (Genomatix, TRANSFAC and PLACE) databases are shown for each TFBS class. [file 1471-2164-13-497-S6.pptx]
